# Hip joint pain in children with cerebral palsy and developmental dysplasia of the hip: why are the differences so huge?

**DOI:** 10.1186/1471-2474-15-96

**Published:** 2014-03-21

**Authors:** Andrzej Grzegorzewski, Marek Jóźwiak, Maciej Pawlak, Tadeusz Modrzewski, Piotr Buchcic, Adrian Masłoń

**Affiliations:** 1 Clinic of Orthopaedics and Pediatric Orthopaedics, Medical University of Lodz, Łódź, Poland; 2 Paediatric Orthopaedics and Traumatology Department, K Marcinkowski Medical University, Poznan, Poland; 3 Department of Physiology, Biochemistry and Hygiene, University School of Physical Education in Poznan, Poznan, Poland; 4 Pathomorphology and Clinical Cytopathology Department, Medical University of Lodz, Lodz, Poland; 5 Clinic of Orthopaedics and Pediatric Orthopaedics, Medical University of Lodz, Lodz, Poland; 6 DAFO, Individual Medical Practice ul, Poselska 10 m. 26, 95-070 Aleksandrow Lodzki, Poland

**Keywords:** Dislocated hip joint, Pain, Cerebral palsy, Developmental dysplasia of the hip

## Abstract

**Backgrounds:**

Non-traumatic hip dislocation in children is most often observed in the course of developmental dysplasia of the hip (DDH) and infantile cerebral palsy. The risk of pain sensations from dislocated hip joint differentiates the discussed groups of patients. Will every painless hip joint in children with cerebral palsy painful in the future?

**Methods:**

Material included 34 samples of joint capsule and 34 femoral head ligaments, collected during open hip joint reduction from 19 children with CP, GMFCS level V and from 15 children with DDH and unilateral hip dislocation. All the children were surgically treated.

The density of nociceptive fibres was compared between the children with CP and DDH, using S-100 and substance P monoclonal antibodies.

**Results:**

More frequent positive immunohistochemical reaction to S-100 protein concerned structures of the femoral head ligaments in children with CP and cartilage losses on the femoral head, when compared to the same structures in children with DDH (p = 0.010). More frequent were found positive immunohistochemical reactions for S-100 protein in the joint capsules of children with cartilage losses (p = 0.031) and pain ailments vs. the children with DDH (p = 0.027). More frequent positive reaction to substance P concerned in femoral head ligaments in CP children and cartilage lesions (p = 0.002) or with pain ailments (p = 0.001) vs. the DDH children.

**Conclusions:**

Surgical treatment of hip joint dislocation should be regarded as a prophylactics of pain sensations, induced by tissue sensitisation, inflammatory process development or articular cartilage defects.

## Background

Non-traumatic hip dislocation in children is most often observed in the course of developmental dysplasia of the hip (DDH) and infantile cerebral palsy (CP) [1-3]. Beside aetiology, these two groups of patients differ between each other with the age, at which hip joint dislocation occurs, the presence of deformities in other elements of musculoskeletal system in children with CP and with an increased risk of congenital malformations (torticollis or clubfoot) in case of children with DDH [4,5]. In children with DDH, pain is never suffered at the infantile age or at initial walking phase, up till the end of the first decade of life. When treatment is not applied, pain is sensed at the earliest by teenage children [6,7]. In children with dislocated hip joint in the course of cerebral palsy, pain sensations were observed in 40%-84% of cases and kids started to complain of pain 3-5 years later after putting diagnosis hip dislocation [8-11]. Literature data indicate that pain more often occurs in children with injured cartilage of the femoral head [12,13].

Hip pain results from the stimulation of free endings of slow-conducting nerve fibres with nociceptive input. This effect can be amplified by biochemical agents, such as bradykinin or histamine, which may activate the endings of nerve fibres, triggering pain, while other agents, such as substance P or prostaglandins, mainly enhance the sensitivity to applied stimuli [14,15]. Moreover, a persistent noxious, mechanical, or thermal stimulation input can be involved in developing or increasing central sensitization and release processes, leading to or supporting chronic pain.

Interestingly enough, pain sensations in children with CP do not result only from degenerative changes in the cartilage in consequence of irritating the exposed, innervated subchondral layer of the femoral head, as pain complaints are also observed in children without significant cartilage lesions [13]. All to-date’s attempts to confirm that children with CP are predisposed to pain sensation from dislocated hip joint have failed to provide unequivocal results [13,14], and there is only a question if an increased expression of substance P and S-100 protein in soft tissues of the joint could be regarded as the cause of pain. An answer to this question could help decide whether preventive, surgical open repositioning of painless hip joints in children with cerebral palsy is a justified procedure and if every painless joint will not generate pain in future.

This question could be solved by a comparison of nociceptors distribution densities and/or factors, predisposing to inflammation development or sustaining in soft tissues of the hip joint in children with its painless dislocation in the course of DDH and in children with painful and painless hip joint dislocation in the course of CP.

## Methods

The studied material included 34 samples of joint capsule and 34 femoral head ligaments, collected during open hip joint reduction from 19 consecutive non-ambulatory children with CP, GMFCS level V (their age range: 5–18 years; the mean age: 9 years 6 months; 10 males; 9 females) with unilateral hip joint dislocation (the migration index >80%) (group 1) and from 15 children with DDH and unilateral hip dislocation (the age range: 2- 4 years; the mean age: 2.6 years; 11 females; 4 males) (group 2).

Children with CP were diagnosed with hip dislocation a few weeks before the surgery. The exact time of dislocation is unknown. All the children were surgically treated because of presence of dislocated hip joint pain or with the goal to prevent pain appearance. The functional purpose of hip surgery was to improve the trunk balance during sitting. During surgery, cartilage destruction levels were assessed by mapping of the cartilage lesions and comparing with the total femoral head surface. Degenerative changes were assessed according to their size as: large (>50% of femoral head surface); medium (25%-50%) or small (<25%).

Primary caregivers were asked in compliance with clinical anamnesis protocol about pain experienced by the children in their care. Caregivers were questioned about the incidence of chronic or incidental musculoskeletal pain localized around the hip joint during the course of performing daily activities and rehabilitation therapy. Pain was assessed on observed idiosyncratic or expressive behavior, such as crying, moaning, agitation, flinching or moving the body parts away. Pain intensity was quantified, using the 11-point Numeric Rating Scale (NRS-11), ranging from 0 = no pain to 10 = severe pain. A subdivision into painless hip joints (NRS-11 from 0 to 2) – Group 1a, and painful hips (NRS-11 from 3 to 10) was made still before clinical analysis to eliminate false-positive indications – group 1b. The NRS-11 borderline value of 2 (slight, occasional pain) was based on the authors’ personal experiences. All the 9 children with cartilage damage felt hip pain. Two of the ten children without cartilage damage felt pain as well (patient No. 7 and No. 10), while in other children without cartilage damage the hip was painless (Table 1).

**Table 1:** Pain and cartilage damage distribution in CP patients

**Patient no.**	**Age**	**Pain yes/no**	**Cartilage damage in %**
1.	11	Yes	30
2.	18	No	0
3.	12	No	0
4.	8	Yes	80
5.	7	Yes	80
6.	11	No	0
7.	8	Yes	0
8.	7	No	0
9.	13	Yes	80
10.	7	Yes	0
11.	12	Yes	60
12.	9	Yes	80
13.	13	No	0
14.	6	No	0
15.	7	Yes	80
16.	5	Yes	80
17.	8	Yes	40
18.	7	No	0
19.	11	No	0

Children with DDH were not diagnosed with the hip dislocation ultrasound screening program because their parents failed to attend but, at the age from 6 to 8 months, they were non surgically treated till the first year of life or diagnosed later during routine paediatric examination and referred to orthopaedic outpatient clinic. All the children presented with hip joint luxation. Children were independent ambulators with no identified systemic disorders or congenital deformities. None of child patients or their caregivers reported any problems with pain, which would have corresponded to NRS-11.

The samples, collected during surgery, were processed for routine light microscopic observations. The tissue samples were fixed for 24 hours in 4% buffered formaldehyde and embedded into paraffin. Then, 4 μm sections were stained with haematoxylin and eosin (H&E) and examined on an Olympus BX 50 light microscope. In the H&E-stained specimens, the number of blood vessels was evaluated in 10 visual fields. The specimens without visible blood vessel were labelled 0, the specimens with a small number of vessels – as 1, those with the average number of vessels – as 2 and, finally, the specimens with a big number of vessels as 3.

The study was approved by the Bioethics Committee of the Medical University. Written informed consent for participation in the study was obtained from children’ parents or guardians.

### Immunohistochemistry

An immunohistochemical study was performed, using the rabbit anti-substance P polyclonal antibody (Chemicon International, Inc.) and the S-100 protein mouse monoclonal antibody (Novocastra, UK). Paraffin sections were mounted onto superfrozen slides and deparaffinised. After re-hydration, the sections were for 5 minutes reacted with 3% hydrogen peroxide in distilled water and rinsed in Tris buffered saline (TBS). Afterwards, the slides were overnight incubated in a moist chamber with the rabbit anti-substance P antibody in 1:2000 dilution and at 4°C. Then, the sections were rinsed in TBS and a DAKO LSAB+/HRP Universal Kit (DAKO A/S, Glostrup, Denmark) was used, according to the manufacturer’s instructions. Positive immunoreactivity was visualized with DAB as chromogen. The sections were washed in distilled water, counter-stained with H&E and coverslipped with DPX mounting medium. Negative controls were carried out by incubation in the absence of the primary body – the results being always negative. A similar procedure was performed with the S-100 protein mouse monoclonal antibody (Novocastra, UK) – (the slides were incubated with S-100 in 1:25 dilution for 60 minutes).

### Assessment method of SP immunopositive fibres

The slices were semi-quantitatively examined for substance P expression, showing nerve fibres, counted in 10 visual fields (lens magnification: ×40). The numbers of immunoreactive fibres were graded as follows:

0 - lack of nerve fibres with substance P expression

1 - one or more immunopositive nerve fibres with substance P expression

In each case, the arithmetic mean of SP immunopositive fibres was assessed for the ten visual fields. A similar procedure was performed with S-100 protein.

The density of nociceptive fibres, exposed by antibodies against Substance P (SP) and S-100 protein and the vascularity of specimens were evaluated in children with DDH and CP with painful and painless hip joints.

Statistical analysis – the U Mann-Whitney test was used.

## Results

The obtained results indicate that the statistically significantly more frequent positive immunohistochemical reaction to S-100 protein concerned structures of the femoral head ligaments in children with CP and cartilage losses on the femoral head, when compared to the same structures in children with DDH (p = 0.010). Also more frequent were found positive immunohistochemical reactions for S-100 protein in the joint capsules of children with cartilage losses (p = 0.031) and pain ailments vs. the children with DDH (p = 0.027). Similarly, the statistically significantly more frequent positive reaction to substance P concerned structures of the femoral head ligaments in children with CP and cartilage lesions (p = 0.002) or with pain ailments (p = 0.001) vs. the children with DDH. An analysis of the vascularisation degree of the femoral head ligament and of the joint capsule did not show any statistically significant differences between patients of either DDH or CP group, see Table 2 for detailed data.

**Table 2:** Correlation-DDH and CP group

		**CP (n = 19)**				
		**Painless (n = 8)**	**Painfull (n = 11)**	**Without cartilage damage (n = 10)**	**With cartilage damage (n = 9)**	
**Teres ligament**	**Blood vessels number**	Z = 0,78, p = 0,437	Z = 1,29, p = 0,198	Z = 0,09, p = 0,930	Z = 0,83, p = 0,408	**DDH (n = 15)**
	**S-100 (number of positive structures)**	Z = 1,06, p = 0,289	Z = 1,95, p = 0,052	Z = 1,31, p = 0,190	Z = 2,59*, p = 0,010*	
	**Substance P (number of positive structures)**	Z = 0,70, p = 0,482	Z = 3,29*, p = 0,001*	Z = 0,000, p > 0,999	Z = 3,12*, p = 0,002*	
**Joint capsule**	**Blood vessels number**	Z = 1,48, p = 0,140	Z = 1,57, p = 0,116	Z = 1,54, p = 0,124	Z = 1,51, p = 0,131	
	**S-100 (number of positive structures)**	Z = 1,06, p = 0,290	Z = 2,21*, p = 0,027*	Z = 0,25, p = 0,806	Z = 2,16*, p = 0,031*	
	**Substance P (number of positive structures)**	Z = 1,64, p = 0,101	Z = 1,42, p = 0,157	Z = 1,17, p = 0,243	Z = 1,89, p = 0,060	

*results statistically significant.

Almost all the children with CP and cartilage defects demonstrated positive immunohistochemical reactions to S-100 protein, substance P or to both studied markers in the articular capsule and/or in the round ligament (Table 3).

**Table 3:** S-100 protein and Substance P distribution in CP patients and cartilage damage

**Patient no. (with cartilage damage)**	**Teres ligament**		**Joint capsule**	
	**S100**	**Substance P**	**S100**	**Substance P**
1.	+	+	-	-
4.	-	+	+	-
5.	+	+	+	+
9.	+	+	-	-
11.	+	+	+	+
12.	+	+	-	-
15.	+	+	-	-
16.	+	+	+	-
17.	-	-	-	+

**Figure 1:**
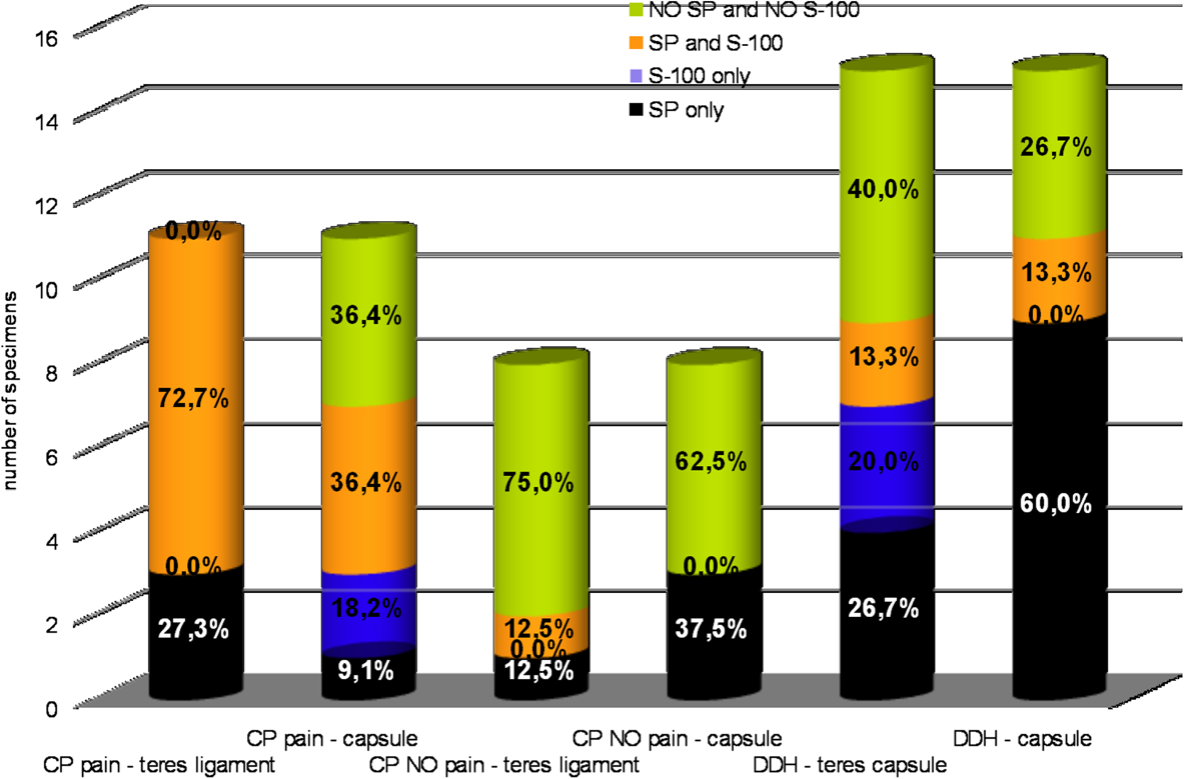
S-100 protein and substance P percentage distribution in the teres ligament and the capsule in groups of CP and DDH patients.

In our study, pain was found in 57.9% of the CP children but not in the DDH patients. 11 CP patients with pain demonstrated higher SP and S-100 densities, when compared to CP patients without pain. In the pain-suffering CP children, both proteins, i.e., S-100 and SP, were present in the teres ligament and in the capsule, 72.7% and 36.4%, respectively. Moreover, only in 3 cases was SP immunoreactivity in the joint capsule found unaccompanied by S-100 (Figure 1).

## Discussion and conclusions

Clinical and radiological symptoms of dislocated hip joint in DDH and CP are almost identical. Even surgical treatment methods of both groups are almost the same. However, the clinical practice shows that pain sensations of dislocated hip joint are more frequently observed in children with CP, which are born with normally developed hip joint, while the loss of contact between articular surfaces is most often observed in these children at the age after 3-5 years [10,16,17].

Numerous studies, concerning the distribution of nociceptive endings in the anatomical structures of various joints and the attempts to analyse their role in pain induction, have already been described in the literature [18-21]. However, there has, so far, been no comparison of nociceptor distribution in hip joint structures of patients with hip joint dislocation in the course of cerebral palsy and developmental dysplasia of the hip or a proper analysis, explaining the effect of differences in this distribution on the occurrence of joint pain sensations.

In our studied groups, pain occurred in more than a half of the patients with cerebral palsy, while such sensations were not observed in the patients with DDH. In all the children with cerebral palsy and cartilage defects, positive immunohistochemical reactions were found for S-100 protein, substance P or for both studied markers in the joint capsule and/or the femoral head ligament. In comparison with patients with hip joint dislocation in the course of developmental dysplasia, a positive immunohistochemical reaction to S-100 protein more frequently concerned structures of the femoral head ligament in children with CP and cartilage defects on the femoral head. Positive immunohistochemical reactions to S-100 protein were also more frequent in the joint capsules of children with CP and cartilage defects and pain sensations than in children with DDH. It was also found that a positive immunohistochemical reaction for substance P concerned more often the structures of the femoral head ligament in children with CP and cartilage defects or pain sensations, when compared to children with DDH.

Saxler et al. observed a 2.5× higher level of immunopositive nerve fibres for substance P and CGRP in structures of osteoarthritis hip joint vs. a control group [20]. Similarly in our studies, a significantly higher content of immunopositive fibres for S-100 protein was found in the femoral head ligament and in the articular capsule of patients with CP, when compared with preparations from the patients with DDH.

The relationship, observed between the occurrence of substance P and S-100 protein and pain intensity in the groups of children with cerebral palsy and with DDH , unequivocally confirmed that pain in the previous group was associated with an increased expression of both these factors. It was particularly distinctive in the femoral head ligament, while less in the joint capsule. In patients, suffering from pain, both markers were higher vs. the group with DDH, in which no pain was observed. Also femoral head cartilage defects in the group of patients with CP were associated with a higher expression of both markers, both in the joint capsule and in the femoral head ligament. These differences emphasise the specific role of inflammatory process, present in defective tissues. This hypothesis was confirmed by authors, suggesting that proteins of the S-100 family, released into the extracellular environment, could play the role of a pro-catabolic and pro-inflammatory factors which stimulate cell proliferation and joint cartilage degradation [22,23].

The results of our studies have also indicated a new, important aspect, concerning possible predispositions to hip joint pain in children with CP, depending on the density of nociceptor layout. In 8, out of eleven (72.7%) cases of children with CP, suffering from hip joint pain, there was a simultaneous increase in the number of immunopositive fibres for substance P and S-100 protein in the femoral head ligament. Pain intensity seems then to be related to concomitant localisation of substance P and S-100 protein, especially in the femoral head ligament. In the group of children with DDH, the lack of pain sensations correlate well with only limited, colocalisation of substance P- and S-100 protein-immunopositive fibres (13.3%).

The aetiology of pain sensations in the group with CP does not provide any clear indication why pain, associated with hip joint dislocation, occurs in some patients only. In histopathological studies, free nerve endings with nociceptive function were identified in all the joint structures except the articular cartilage. Despite the lack of innervation and vascularisation, cartilage defects induce pain sensations of, so far, non defined aetiology. It is very well possible that an accumulation of microtraumas may lead to cartilage wear, as well as to osteoporotic fractures [24]. Also a gradual loss of the articular cartilage may lead to pain sensation and reduced articular mobility, resulting from an exposure of nerve endings in the subchondral layer.

It cannot be excluded that an increased density of the nerve fibres indicating expression for substance P, may be a consequence of direct and indirect inflammatory processes that take place in degenerative and defective joint structures around the cartilage [25,26].

Some authors have found immunopositive fibres for substance P in articular structures, such as the synovial membrane, the meniscus and ligands [26]. The presence of substance P-containing nerve fibres in the femoral head ligament, may suggest their important, nociceptive role in the hip joint, supportive for the development of pathophysiological processes in the femoral head ligament and the joint capsule. Their intensity and scope demonstrate among others Witoński and Wagrowska-Danilewicz [27]. They found that the number of P-positive nerve fibres was not constant and might undergo a certain increase during a 4-month period from the injury. Also in our groups of patients, the time period of irritating the femoral head ligament and joint capsule tissues was different, what resulted from the already mentioned changes in the hip joint in the course of developmental dysplasia, which have their beginning already in the foetal life.

The question still remains unanswered, whether there is any relationship between the occurrence of hip joint pain in children with CP and the density of nociceptors. The results of studies by Maslon et al. [13], as well as of studies by other authors [20], confirm that there is such a relationship, moreover indicating a specific relationship between the density of substance P-positive fibres and pain occurrence. In hip joint dislocation in the course of spasticity as well as in the presence of other aetiological factors, leading to pain induction by the hip joint, an increased density of nociceptive fibres is closely related with pain intensity. The whole set of complex activities, including the activation of nerve fibres, immunopositive for substance P and S-100 protein, initiates important cellular processes which, in turn, trigger a number of secondary, intracellular transmitter systems, especially protein kinases [26].

So huge clinical differences between CP and DDH groups with dislocated hip joint, expressed in the frequency of pain, seems to taking its source in tissue sensitization and inflammatory process or articular cartilage defects. At present, the only way to stop the adverse biological processes is early surgical treatment.

## Competing interests

The authors declare that they have no competing interests.

## Authors’ contributions

AG - conception of the study, manuscript, collecting material. AM - conception of the study, manuscript, collecting material. MJ - conception of the study, manuscript, statistical analysis. TM – Immunohistochemical staining and analysis. MP - conception of the study, manuscript, statistical analysis. PB – Manuscript. All authors read and approved the final manuscript.

## Pre-publication history

The pre-publication history for this paper can be accessed here:

http://www.biomedcentral.com/1471-2474/15/96/prepub
